# Single posterior approach for circumferential debridement and anterior reconstruction using fibular allograft in patients with skipped multifocal spinal tuberculosis

**DOI:** 10.1186/s13018-022-03372-2

**Published:** 2022-11-16

**Authors:** Yen-Chun Chiu, Shih-Chieh Yang, Yu-Hsien Kao, Yuan-Kun Tu

**Affiliations:** Department of Orthopedic Surgery, E-Da Hospital/I-Shou University, No. 1, E-Da Road, Kaohsiung City, 82445 Taiwan

**Keywords:** Fibular allograft, Single posterior approach, Skipped infection, Spinal tuberculosis

## Abstract

**Background:**

Skipped multifocal spinal tuberculosis (TB) is an atypical presentation of spinal TB. Surgical treatment for these unusual cases remains a challenge for spine surgeons. In our institute, we used single-stage circumferential debridement and anterior reconstruction with fibular allograft followed by posterior instrumentation through posterior-only approach for these patients. This study aimed to determine the efficacy and feasibility of this technique.

**Methods:**

Twelve patients with skipped multifocal spinal TB who received our treatment method from January 2012 to June 2020 were enrolled in this study. The visual analog score (VAS), laboratory data, comorbidities, complications, and neurologic status based on Frankel scale were recorded. The patients’ clinical conditions were evaluated based on modified Brodsky’s criteria and Oswestry Disability Index (ODI).

**Results:**

All the patients were infection free at the end of the treatment. The average VAS score was 7.5 (range, 7–8) before surgery and decreased to 2.1 (range, 1–3) one year postoperatively. No one experienced any severe complications such as neurologic deterioration, fixation failure, or bone graft dislodgement. Out of the three patients requiring debridement surgery, two had wound infection and one had seroma formation. The ODI score improved from 76.8 (range, 70–84) preoperatively to 25.5 (range, 22–28) one year after surgery. All patients achieved good or excellent outcome based on modified Brodsky’s criteria one year postoperatively.

**Conclusions:**

In our study, the patients could achieve a good clinical outcome. This technique could be an alternative for patients with skipped spinal TB.

## Introduction

Spinal tuberculosis (TB), also known as Pott’s disease, refers to vertebral osteomyelitis and intervertebral discitis caused by TB infection which is often associated with significant morbidity and could even lead to severe functional impairment [1]. Management for these patients is still a challenge for clinicians because the spine structure could be severely destroyed by TB infection resulting in spinal instability, kyphotic deformity, neurologic injury, and even paralysis [2]. There is still no standard protocol for treating such patients [3].

Conservative management with anti-TB chemotherapy is considered as the first line of treatment [3]. Most patients can be cured by a strict and standardized treatment protocol using anti-TB medications. However, surgical intervention is indicated in patients with progressive neurologic deficit, spinal instability, extensive paravertebral or epidural abscess, and severe spine deformity [4]. The goal of surgery is not only extensive debridement for the infective tissue but also decompression of neurologic elements, correction of deformity, reconstruction of spinal structure, and restoration of stability for early mobilization. Although numerous operative methods have been proposed for such patients, there are still controversies as to which one is better [5].

Skipped multifocal spinal TB is defined as at least two separate levels of spinal infection which is considered as an atypical manifestation of spinal TB [6]. A higher incidence of neurologic deficit resulting in an increased requirement for surgical interventions was reported [6]. The multifocal distribution of infection foci could also increase the complexity during operation. It is troublesome to manage all infective levels through an anterior approach, and multiple incisions may be required. Posterior decompression and instrumented fusion had been reported to treat spine TB successfully and seems to be an alternative to manage this atypical condition [7, 8]. However, it is difficult to achieve a good reduction of kyphotic deformity, ensure complete eradication of infective tissue over the anterior column, and enable comprehensive decompression of neurologic elements as the compression could be located either anteriorly or posteriorly. Furthermore, the stability may be insufficient without using a strut bone graft or body spacer for anterior support. Until now, there are only few reports mentioning about the surgical management of patients with noncontiguous spine TB and no consensus exists [6, 9–11]. In our institute, we used the single-stage technique through a posterior-only approach with circumferential decompression and anterior column reconstruction using fibular allografts to treat patients with skipped spinal TB over the thoracolumbar spine since 2012 [12]. The purpose of this study was to evaluate the feasibility and efficacy of this technique through a retrospective review of patients’ medical records who underwent surgery.

## Materials and methods

### Patients

The study population comprised 96 consecutive patients who underwent surgical treatment for spinal TB over thoracolumbar spine from January 2012 to June 2020 in our institute. Of these, 13 were diagnosed with skipped spinal TB. One patient died seven months after the index surgery due to myocardial infarction and was excluded as he could not complete a one-year follow-up. After the study was approved by the institutional review board and informed consent was obtained from each patient, medical records of remaining twelve patients’ were reviewed retrospectively.

**Fig. 1:**
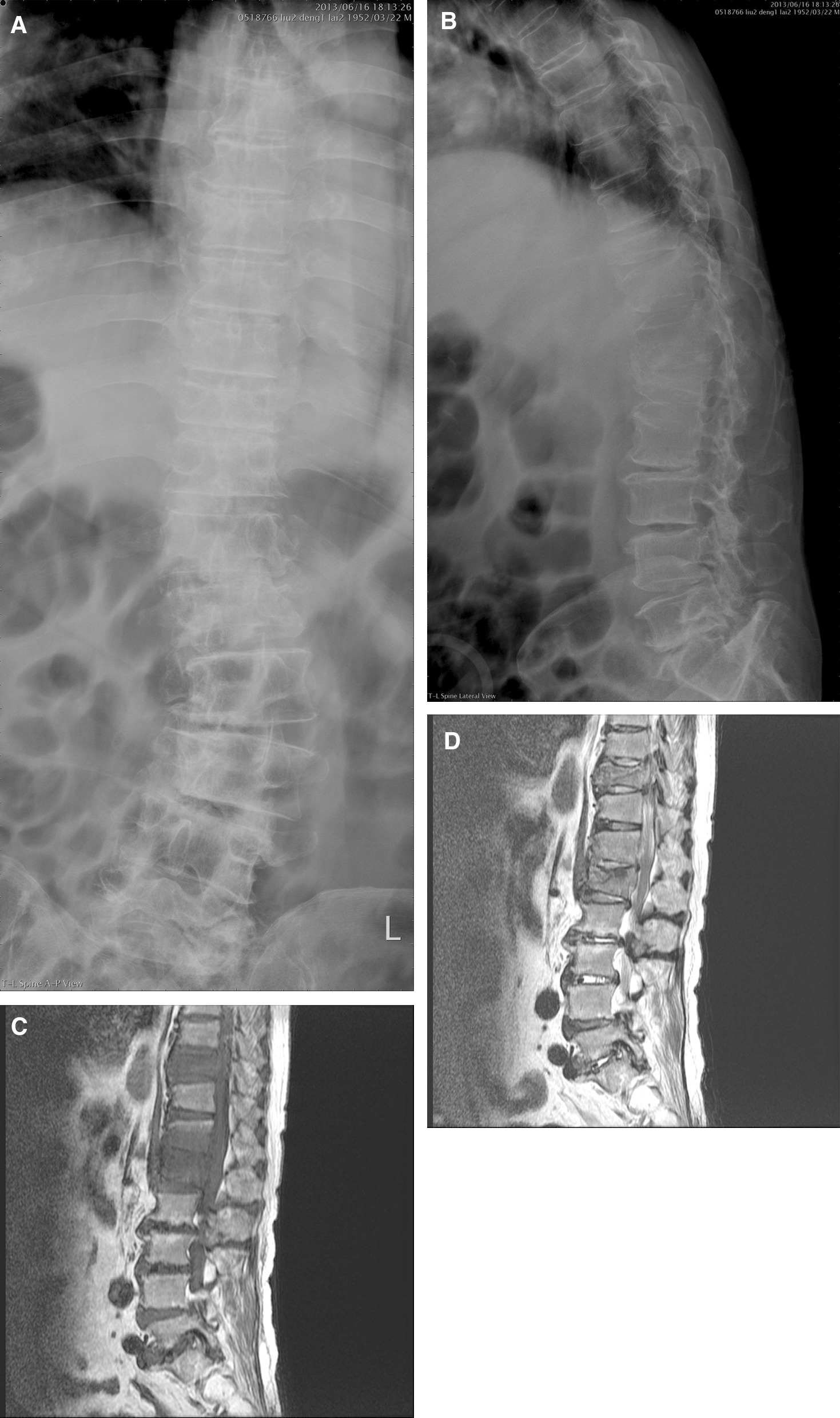
A 60-year-old male patient came to our outpatient clinic due to progressive back pain and intermittent onset of low-grade fever for more than one month. Anteroposterior (**A**) and lateral (**B**) view radiographs show vertebral body collapse of T10 and L1. T1-weighted (**C**) and T2-weighted MRI (**D**) reveals skipped spinal TB over T10 and L1 levels

All 12 patients had symptoms of progressive back pain which could not be controlled by painkillers and bracing. Neurologic deficit with Frankel C to D was noted in 10 cases. The diagnosis was based on the clinical presentation, pathologic reports, microbiology laboratory results, and magnetic resonance imaging of spine (Fig. 1). Conservative methods were prescribed for each patient as first line of treatment. Single-stage surgery through posterior approach was performed after the conservative methods failed. Radiographic assessment was carried out before and after surgery, at the 1-, 3-, 6-, and 12-month visit after discharge, and every year thereafter. The inflammatory markers which included C-reactive protein (CRP) and erythrocyte sedimentation rate (ESR) were checked regularly to monitor the status of infection control until spinal TB was cured. All 12 enrolled patients were followed up for at least 18 months after undergoing the surgical procedure.

### Surgical technique

#### Exposure and posterior stabilization

**Fig. 2:**
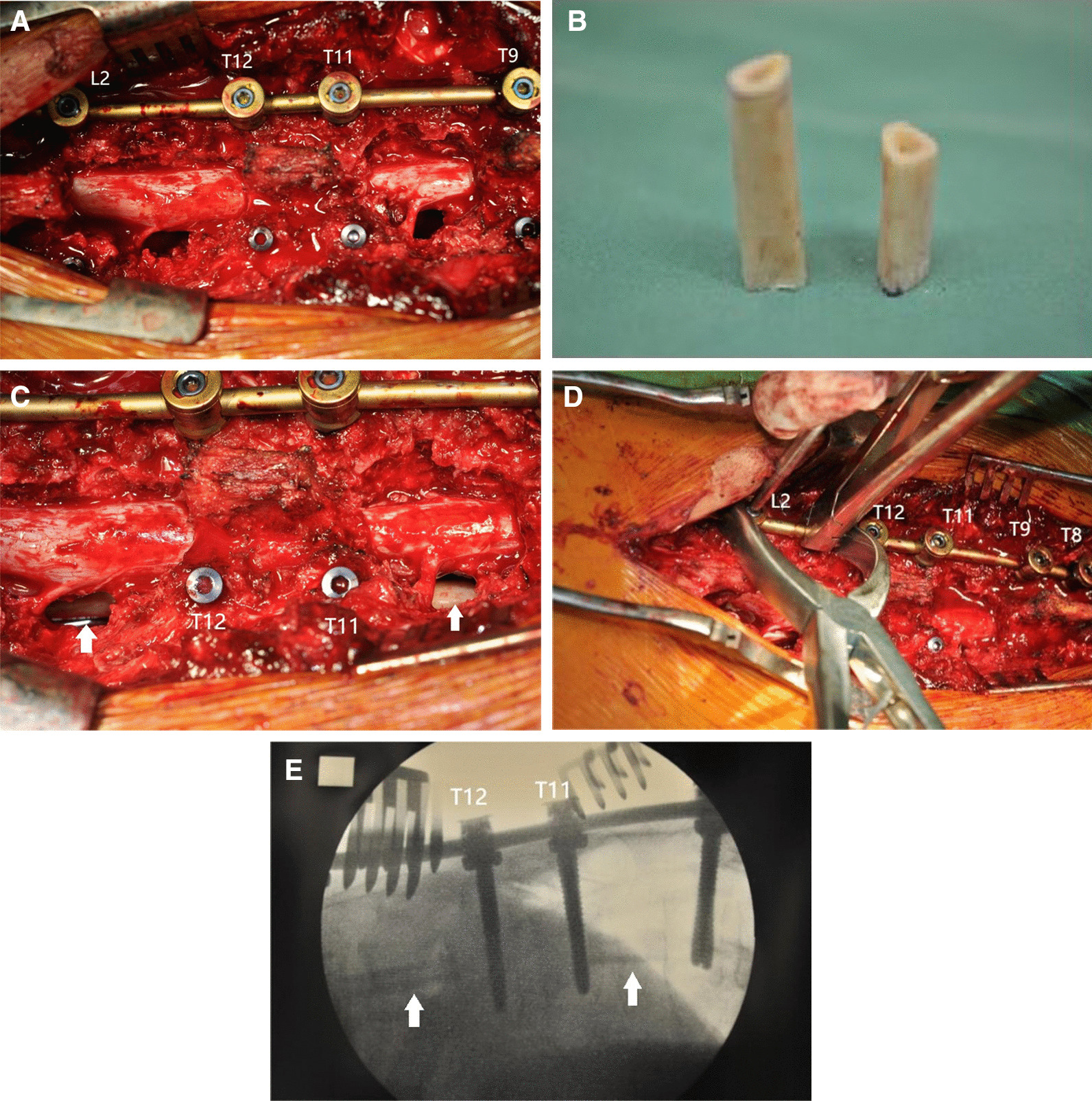
The posterior complex was exposed through posterior midline incision. Transpedicular screws were inserted, and one rod was placed temporarily to provide adequate stability during surgery. Circumferential debridement could be achieved through transpedicular approach, as shown in (**A**). Preparation of adequate length of allogenic fibular bone grafts for anterior column reconstruction (**B**). Introduction of the bone grafts through the routes between nerve roots without sacrificing any neurologic elements (**C**). Compression of the adjacent instrument to obtain a good contact between endplate and allograft, as well as a better alignment (**D**). Intraoperative fluoroscopy to check the position of bone grafts and instruments (**E**)

After induction of the general anesthesia, the patient was placed in the prone position on pads. The patients’ trunk, face, and extremities were also positioned properly to avoid occurrence of pressure sores. Either one extended or two midline incisions according to the infection levels was made. We also used intraoperative C-arm fluoroscopy to identify the exact level. The posterior complex was exposed followed by inserting transpedicular screws into two levels above and two levels below the infective vertebrae. For patients with the 5th lumbar or sacral involvement, iliac screws were introduced to achieve an adequate fixation. One rod was placed temporarily for keeping the spine stable to prevent further neurologic injuries by vibration during surgery (Fig. 2A). The temporary rod was placed on the contralateral side of planned allograft insertion, which was determined by the findings of preoperative imaging studies and neurologic examination, as it could influence the transpedicular debridement and access for allograft insertion. Sometimes, extensive debridement is indicated and, in this case, bilateral transpedicular debridement was required. In this situation, another rod on the opposite side was placed followed by releasing the previous fixed one after we finished the procedure on one side. Therefore, decompression and debridement on both sides could be achieved without creating undesired vibration.

#### Debridement and decompression

Bilateral fasciectomy and laminectomy were performed to expose the involved pedicles and neurologic elements. Removing all the infective tissue and unhealthy bone for circumferential debridement was achieved using transpedicular approach through the interval between the nerve roots. Although sacrificing or ligating thoracic nerve roots seem to be acceptable, it was unnecessary during our procedure because the working space was enough. The anterior cortex or anterior longitudinal ligament was preserved to provide protection to anterior vessels, diaphragm, visceral organs, or other vital structures.

#### Anterior column reconstruction

After circumferential debridement and complete decompression for neurologic elements was achieved, the length of bony defect was then measured with Kirschner wire under fluoroscopy. Adequate length of fibular allograft was prepared, and multiple drill holes were made on it to increase the rate of bone incorporation between the bone graft and the vertebral body (Fig. 2B). The fibular bone graft was then inserted through the space between the nerve roots anteriorly. After the allogenic bone graft was placed adequately, both rods and link were applied for immediate stability (Fig. 2C). In order to obtain a good contact between endplate and allograft, as well as to achieve a better alignment, we compressed the adjacent instrumentation (Fig. 2D, E).

#### Perioperative care

A multidisciplinary team care involving spine surgeon, radiologist, pulmonologist, and infectious disease physician was initiated preoperatively. Chest X-ray of each patient was obtained to find out is there any pulmonary TB. A standardized and strict treatment protocol with anti-TB drugs was initiated once the diagnosis was confirmed [13]. The types and doses of medication were determined based on pulmonologists and infectious disease physicians’ recommendations and then adjusted according to the response after treatment and results of culture. The anti-TB drugs were stopped when the patient fulfilled at least a standard 9-month treatment course and there were no signs of recurrent severe back pain or elevated inflammatory marker levels, which may indicate an incompletely treated spine TB. After surgery, regular aseptic wound care and adequate painkillers were prescribed. The hemodynamic status, wound condition, and neurologic status were regularly checked. Patients were mobilized to wheelchair as soon as possible; ambulation training was arranged as long as the pain and neurologic status could be tolerated. A Taylor’s brace or body jacket was arranged for each patient. The duration of TB medication was based on patient’s clinical presentation, and at least nine months of treatment course was carried out. All the enrolled patients were followed up for at least 18 months at the outpatient department.

#### Outcome assessment

**Fig. 3:**
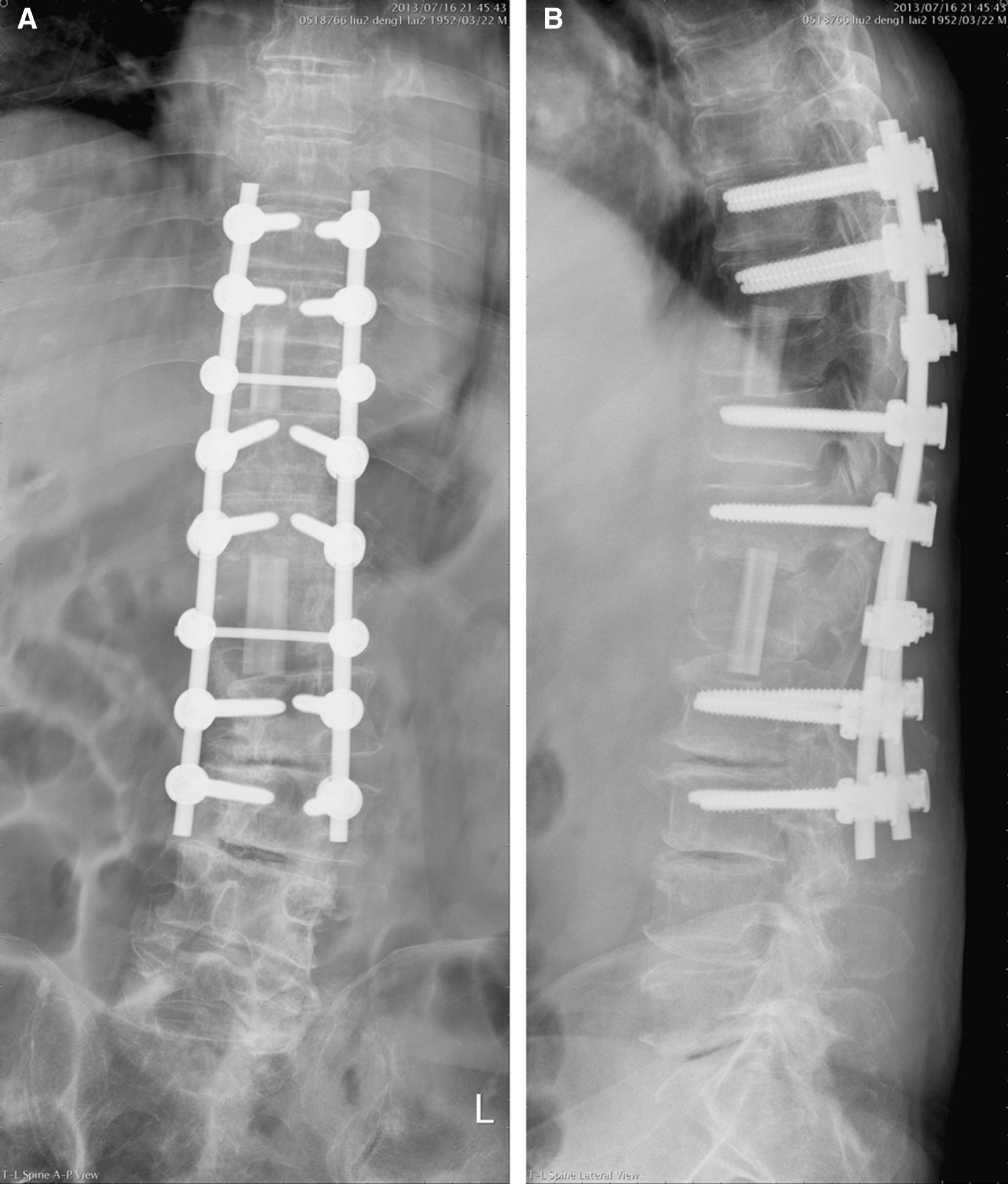
The postoperative anteroposterior (**A**) and lateral (**B**) radiographs reveal an acceptable alignment, which was restored with adequate length of allogenic fibular bone grafts and posterior instrumentation

Radiographic examinations were carried out regularly to evaluate recovery and to find out any undesired complications such as implants failure, obvious nonunion or pseudoarthrosis, dislodgement of allograft, or loss of fixation (Fig. 3). Clinical outcomes were assessed by asking the patients to evaluate their pain on a visual analogue scale (VAS, using a scale of 0–10; 0 meaning no pain and 10, the worst pain possible) and on the basis of pain, activity, and analgesics requirement to determine the modified Brodsky’s criteria, which were categorized as poor, fair, good, and excellent. The functional scores based on Oswestry Disability Index (ODI) were also evaluated. The VAS, modified Brodsky’s criteria, neurologic status based on Frankel scale, and ODI scores before surgery were compared with those before discharge, and 1 year later.

## Results

There were seven men and five women, whose average age was 62.3 years (range, 46–79 years) (Table 1). All the operations were performed by a spine team headed by a senior surgeon (YSC). The most prominent clinical sign of skipped spinal TB was severe back pain, and the VAS decreased from an average of 7.5 (range, 7–8) preoperatively to 3.3 (range, 3–4) before discharge, which further improved to 2.1 (range, 1–3) 1 year postoperatively. Finally, all the patients were cured from TB infection. No one experienced any severe complication such as neurologic deterioration, fixation failure, or bone graft dislodgement. No obvious nonunion or pseudoarthrosis of fibular allograft was detected on radiographic examinations. Neurologic deficits with Frankel C to D were noted in 10 patients preoperatively. One year postoperatively, only two patients still had abnormal neurologic functioning with Frankel D (Table 2). Two patients suffered from poor wound healing and local infection. Hence, they received surgical debridement and wound repair followed by antibiotics. Another patient received debridement surgery because of seroma formation. The ODI score improved from 76.8 (range, 70–84) preoperatively to 30.7 (range, 22–38) before discharge, and 25.5 (range, 22–28) one year after surgery. All patients achieved a good or excellent outcome based on modified Brodsky’s criteria at one year postoperatively (Table 3).

**Table 1:** Patients’ demographic data

Case No	Age (years)	Sex	Infection level	Instrumented level	Comorbidity	Highest ESR before Surgery (mg/dL)	Follow-up (month)	Complication
1	66	M	T9-10/L2-3	T7-L5	No	50	23	No
2	60	M	T10, L1	T8-L3	DM, HTN, Hepatitis C, CAD	39	48	No
3	73	F	T12-L1/L5-S1	T9-Iliac	No	45	56	No
4	50	M	T7/L3	T5-T9/L1-L5	HTN, CAD, Gastric ulcer	50	44	No
5	58	M	T11-L1/L4	T8-S1	No	35	35	Seroma formation
6	75	F	L1, L4	T11-S1	HTN	26	18	No
7	57	M	L2/L5	T11-Iliac	HTN, DM	70	26	No
8	63	F	T11/L1	T9-L3	No	47	43	No
9	46	M	T7-8/L4	T5-T10/L2-Iliac	Drug addition, HIV	55	32	Wound infection and poor healing
10	79	F	T10/L4	T8-S1	DM, HTN	40	26	No
11	48	F	L2/L4	T12-S1	Rheumatoid arthritis	57	28	Wound infection and poor healing
12	73	M	T8-9/T12	T6-L2	No	40	22	No

*M* Male, *F* Female, *T* Thoracic spine, *L* Lumbar spine, *S* Sacral spine, *DM* diabetes mellitus, *HTN* hypertension, *CAD* coronary artery disease, *HIV* human immunodeficiency virus, *ESR* erythrocyte sedimentation rate

**Table 2:** The improvement of visual analog scale and Frankel scale before surgery, before discharge (postoperative), and one year later

Case number	Pre-op VAS	Post-op VAS	1 yr VAS	Pre-op FS	Post-op FS	1 yr FS
1	8	3	2	C	D	D
2	8	3	2	D	E	E
3	7	4	1	D	D	E
4	8	4	3	D	D	E
5	7	3	2	D	D	D
6	7	4	3	C	E	E
7	8	3	1	E	E	E
8	8	3	2	D	E	E
9	7	3	3	C	D	E
10	7	3	2	E	E	E
11	8	4	2	D	D	E
12	7	3	2	D	E	E

*Pre-op* preoperative; *Post-op* postoperative; *1 yr* one year later; *VAS* visual analog scale: 0 means no pain, and 10 means the most pain possible; *FS* Frankel scale: *A* complete paralysis; *B* sensory function only below the injury level; *C* incomplete motor function below injury level; *D* fair to good motor function below injury level; and *E* normal function

**Table 3:** The improvement of modified Brodsky criteria and Oswestry Disability Index before surgery, before discharge (postoperative), and one year later

Case number	Pre-op MBC	Post-op MBC	1 yr MBC	Pre-op ODI	Post-op ODI	1 yr ODI
1	P	G	G	78	26	24
2	P	E	E	80	22	22
3	F	G	E	76	30	24
4	P	G	G	72	28	24
5	F	G	G	72	32	28
6	P	G	G	76	30	28
7	P	G	E	78	34	24
8	P	G	G	82	32	28
9	P	F	G	84	38	26
10	F	G	G	76	32	28
11	P	F	G	70	36	26
12	P	G	E	78	28	24

*Pre-op* preoperative; *Post-op* postoperative; *1 yr* one year later; *MBC* modified Brodsky criteria: *P* poor, *F* fair, *G* good, *E* excellent; and *ODI* Oswestry Disability Index

## Discussion

Spinal TB is defined as a chronic infection caused by *Mycobacterium tuberculosis* which most commonly affects the thoracolumbar junction but the whole spine could be involved [2–5]. It is usually caused by hematogenous spread of offending pathogen from the primary infection site, most common in pulmonary region or genitourinary system [14], to the vasculature of vertebral bodies. As the reputation of a great mimicker in medicine, the clinical pictures of spinal TB are varied with a wide range from insidious back pain to severe spinal deformity, neurologic deficit, and even paralysis [1–3]. The non-specific manifestation makes timely diagnosis, the most crucial part for successful treatment and avoiding complications, remain a challenge for clinicians. Typically, the presentations in images involve destruction of the vertebral bodies and intervertebral disk space, collapse of the spinal structures, and anterior wedging leading to kyphosis and gibbus deformity. However, many atypical features have been reported in the literature.

Pande et al. presented a new classification of atypical spinal TB and described that noncontiguous lesion is one of the atypical manifestations [15]. This atypical condition is not uncommon with the reported incidence ranging from 1.1 to 16.3% in the literature [16]. Kaila et al. even reported an incidence up to 71.4% by reviewing the whole spine magnetic resonance imaging (MRI) of 14 patients [17]. Several possible reasons had been mentioned to explain the high incidence of skipped spine TB. First, it may be related to embolic spread of bacteria to multiple levels of vertebrae which is similar to the phenomenon of multiple metastasis in tumor cases [18]. Second, Batson’s paravertebral venous plexus, a valve-less system in the vertebrae, allows the spread of infection along it and is considered as a reason in developing noncontiguous lesions [19]. However, this is still controversial and the real reason remains unclear.

Conservative management with multiple anti-TB drugs is the mainstay in managing spinal TB because varying categories of bacilli could exist. It could also reduce the instance of drug resistance. According to the recommendations of WHO, four drugs: isoniazid, rifampicin, pyrazinamide, ethambutol, or streptomycin, are administered for two months in the initiation phase followed by two drugs—isoniazid and rifampicin, for seven months in the continuation phase. Kanamycin, amikacin, capreomycin, levofloxacin, etc., are considered as second-line drugs and should be used carefully as they are expensive and have more side effects [13]. However, the adequate treatment should be individualized and should be based on patients’ conditions, response to treatment, and results of culture. In our case series, we followed a treatment protocol with multidisciplinary team care as it could improve the outcomes [20, 21]. Eventually, all the patients could be cured from spinal TB.

Surgery for spinal TB is usually reserved for patients with (1) refractory disease, (2) severe kyphosis, (3) pan-vertebral lesions, (4) progressive neurologic deficit, and (5) clinical deterioration [1–5]. The operation is commonly accessed through the anterior retroperitoneal or transthoracic approach for debriding the infective tissue and decompressing neurologic elements comprehensively. Then, the anterior column was reconstructed using autograft, allograft, or vertebral body spacers. For obtaining an immediate stability, supplemental posterior instrumentation after anterior column reconstruction in a single- or two-stage manner could be carried out through additional posterior approach [22, 23]. This combined surgery is the most secure procedure for patients with severe deformity, instability, and neurologic deficit. However, the major concerns for this combined approach are (1) longer operative time, (2) greater blood loss, (3) higher risk of complications, (4) greater surgical trauma for patients, (5) the need to perform diaphragm take down and rib cutting, and (6) technically difficult [24]. Due to the above-mentioned reasons, this is not an ideal technique, especially for patients who are older or with multiple comorbidities because it may hurt such fragile cases.

To decrease the morbidities and disadvantages related to the combined approach, the technique through a posterior-only approach in single-stage manner had been proposed. Circumferential debridement, decompression of neurologic elements, and even total or subtotal corpectomy followed by anterior reconstruction could be carried out through the posterolateral transpedicular approach without sacrificing the nerve roots. The blood loss, operative time, surgery-related complications, and length of hospitalization can be reduced. In addition, most of the surgeons are more familiar with the posterior instead of anterior approach, and revision surgery is much easier if necessary. This technique had been used to treat a wide range of spinal disorders and resulted in a satisfactory outcome [25–27].

Autograft, allograft, or vertebral body spacer could be used for anterior column reconstruction after circumferential debridement through transpedicular approach. Different pros and cons exist in each choice, and there is still no consensus in which one is better [28–30]. Autogenous iliac or fibula strut bone graft is an ideal choice for anterior column reconstruction during spine surgery as it is osteo-inductive and osteo-conductive, biomechanically stable, and biocompatible. However, the major concern of using autogenous strut bone graft is the high incidence of donor site morbidities [31, 32]. The need for creating another wound followed by an additional procedure for harvesting the bone graft can cause discomfort in these fragile patients. An expandable cage is another recommended choice as it could be passed through the route between the nerve roots and then easily be expanded to adequate length [33, 34]. However, it is not an ideal alternative due to the lack of osteo-inductive and osteo-conductive properties. In our case series, we used allogenic fibular bone graft to reconstruct the anterior column. We chose fibula because the size is suitable for inserting it anteriorly through the route between nerve roots. The major advantage of allograft is that it eliminates the need for additional surgical procedure and prevents donor site morbidities. However, there are some disadvantages related to allograft which included accelerated bone resorption, delayed vascular penetration, slow bone formation, and incomplete or delayed graft incorporation. Some authors also had reported a good outcome by using allograft to reconstruct spinal structure [30]. In the present study, none of our patients experienced an allograft-related complication and achieved good outcome.

### Limitations

Although the clinical outcomes are quite satisfactory in our patients, there are still some limitations in this study. First, there were only 12 patients enrolled; the sample size is too small to prove the efficacy and feasibility of using this technique to treat skipped spinal TB. Second, the retrospective nature of our study design lacked randomization. Hence, it was impossible to enroll patients who underwent different treatment methods for subsequent comparison. Third, the infectivity levels and patients’ health conditions are diverse which may influence the analysis. Fourth, computed tomography was not routinely used to evaluate the fusion of fibular allograft due to the policies of our national health insurance. As plain radiographic examinations alone are not enough to assess the union of allograft, some nonunion or pseudoarthrosis may be undetected. Finally, the quality of bone in each patient is unknown because we did not conduct bone mineral density examinations routinely which may affect the outcomes. For these reasons, further prospective randomized studies are required to prove the feasibility and efficacy of the technique.

## Conclusion

Although the current study only included a small sample size, good results could be achieved by using our proposed treatment method. Hence, our technique can serve as a valuable alternative surgical method for treating patients with noncontiguous spinal TB.

## Data Availability

The patients’ raw data can be obtained in our institute.

## Abbreviations

TB: Tuberculosis
EPTB: Extrapulmonary TB
CRP: C-reactive protein
ESR: Erythrocyte sedimentation rate
VAS: Visual analogue scale
ODIP: Oswestry Disability Index
MRI: Magnetic resonance imaging
